# The role of KRT17 in colorectal cancer

**DOI:** 10.18632/aging.203471

**Published:** 2021-08-25

**Authors:** Daisuke Ujiie, Hirokazu Okayama, Koji Kono

**Affiliations:** 1 Department of Gastrointestinal Tract Surgery, Fukushima Medical University School of Medicine, Fukushima, Japan

**Keywords:** KRT17, stage II colorectal cancer, prognostic biomarker, gene expression, immunohistochemistry

Colorectal cancer (CRC) is one of the most common cancers worldwide. Although approximately 30% of patients with CRC are diagnosed with stage II disease, there remains a challenging question of how to best manage those patients after surgery. We have recently identified a promising candidate, the expression of KRT17 (Keratin 17, K17) that may robustly stratify postoperative patients with stage II CRC into high and low risk of disease recurrence [1].

The mainstay of treatment for locoregional disease is surgical resection, and prognostic stratification and therapeutic decisions largely depend on pathological analysis of the resected specimen according to the TNM classification. Stage I patients have particularly good prognosis after surgery alone, whereas stage II and III patients who have undergone potential curative resection may benefit from postoperative adjuvant chemotherapy to reduce the risk of recurrence and cancer death. Since metastatic disease recurrence is thought to arise from occult micrometastases that are present at the time of surgery, the purpose of adjuvant chemotherapy is to eradicate those micrometastases remaining after potential curative resection. Current clinical guidelines from expert groups clearly recommend that patients with stage III (node-positive) disease receive adjuvant chemotherapy as a standard approach, as it demonstrated a 20-30% relative reduction in overall mortality in this setting. As for stage II (node-negative) patients, the benefit of adjuvant chemotherapy remains debatable, because its absolute overall survival benefit was limited for the average patients with stage II disease (3-4% improvement), leading to potential overtreatment but with a risk of severe toxicities [2]. Clinical guidelines suggest consideration of adjuvant chemotherapy for a subset of stage II patients who have high-risk clinicopathological characteristics, although direct evidence is lacking and the definition of high-risk features are variable among guidelines [2,3]. We therefore aimed to develop molecular assays predictive of disease recurrence for postoperative patients with stage II CRC [1]. Using a total of seven independent datasets of resected stage II CRC based on various microarray and RNA-seq platforms, we conducted a step-wise screening and validation of single genes whose expression levels were significantly associated with disease recurrence. The identification and confirmation of the prognostic impact of KRT17 transcript in multiple cohorts was then recapitulated by immunohistochemistry for KRT17 protein using two independent cohorts of resected specimens. Stage II patients exhibiting positive KRT17 staining in tumor had significant poor disease-free survival and it was statistically independent of previously-known high-risk clinicopathological features. Our findings suggest that KRT17 immunohistochemistry may have potential to be used as a prognostic biomarker for the management of stage II patients in the current clinical setting; for patients with positive KRT17 expression, intensive postoperative intervention, including adjuvant chemotherapy with more appropriate surveillance plans, may be considered. Prospectively designed clinical studies in large cohorts of stage II patients are needed to further validate the prognostic performance of KRT17 expression.

Consistent with the prognostic impact of KRT17 expression in CRC, high levels of KRT17 mRNA and/or protein expression have been correlated with aggressive tumor phenotypes and unfavorable survival outcomes in many solid malignancies, including breast, cervical, ovarian, endometrial, urothelial, pancreatic, gastric, esophageal and oral cancers [4–6]. Moreover, tumor promoting functions of KRT17 have been characterized using several in vitro and in vivo models. KRT17 is not expressed in healthy skin, but is rapidly inducible in skin epithelial cells under stress condition and is ectopically expressed by tumor cells that can regulate multiple cellular processes, such as cell proliferation and inflammatory response. In a mouse model of skin tumorigenesis, genetic ablation of KRT17 delays tumor onset and tumor growth, in part through immunomodulatory mechanisms involving a polarization of chemokine expression profiles from a Th1/Th17 to a Th2 character [7]. KRT17 also appeared to promote skin tumorigenesis by interacting with a RNA-binding protein hnRNP K, regulating the expression of multiple chemokine genes, such as CXCR3 ligands CXCL9, CXCL10 and CXCL11 [5]. Furthermore, KRT17 and a transcriptional regulator AIRE colocalize in the nucleus of tumor keratinocytes and bind to NF-κB consensus sequences at the promotor regions of pro-inflammatory genes [6]. In cervical cancer, KRT17 functions as an oncoprotein by binding to a cell cycle inhibitor p27^KIP1^ and facilitating its nuclear export and degradation to promote sustained cell cycle progression [4]. KRT17 activates Akt signaling and is required for oncogenic transformation of Ewing sarcoma [8]. Although those data highlight a myriad of oncogenic roles for KRT17 in the regulation of gene expression, cell proliferation, inflammation and immune response during tumor initiation and growth, the exact mechanisms of KRT17 expression affecting poor prognosis of CRC remain largely undetermined. We suggest that future mechanistic studies, particularly with the use of in vivo genetic tumorigenesis models, are needed to understand the functional role of KRT17 in CRC possibly through the modulation of the tumor immune microenvironment.
